# Effectiveness of short peripheral intravenous catheter educational programmes to improve clinical outcomes protocol for a systematic review

**DOI:** 10.1016/j.mex.2023.102352

**Published:** 2023-08-28

**Authors:** Daniele Privitera, Erika Bassi, Chiara Airoldi, Nicolò Capsoni, Gloria Innocenti, Isabella Santomauro, Alberto Dal Molin

**Affiliations:** a Department of Biomedicine and Prevention, University of Rome Tor Vergata, Rome, Italy; b Department of Emergency Medicine, ASST Grande Ospedale Metropolitano Niguarda, Milan, Italy; c Department of Translational Medicine, University of Eastern Piedmont, Novara, Italy; d Centro Documentazione Biomedica, ASST Grande Ospedale Metropolitano Niguarda, Milan, Italy; e Health Professions’ Direction, Maggiore della Carità Hospital, Novara, Italy

**Keywords:** Training programme, Health professions, Clinical relevance, Infiltration, Dislodgment, Occlusion, Phlebitis, Infection, Protocol for a systematic review

## Abstract

The placement of a short peripheral intravenous catheter (sPIVC) is the most common invasive clinical procedure for patients requiring fluid infusion and multiple blood draws. Phlebitis and infiltration represent the most common catheter-related complications. Occlusions, dislocations, and infections are less frequent. Insufficient knowledge and skills may increase the risk of these complications. This review aims to evaluate the effectiveness of training programmes to reduce sPIVC failure amongst hospitalised patients. We will search PubMed, Embase, CINAHL, Cochrane Central Register of Controlled Trials (CENTRAL), Cochrane Vascular Specialized Register through the Cochrane Register of Studies, and Google Scholar. We defined the search query using the PICO framework (Participants: health professionals; Intervention: training programme; Comparison: No training programme; Outcomes: all-cause catheter failure). We will include experimental studies evaluating an educational programme to reduce early sPIVC failure amongst hospitalised patients. Two reviewers will independently screen studies for inclusion, extract data, and perform the risk of bias assessment using the Cochrane Effective Practice and Organisation of Care Risk of Bias tool for randomised controlled trials. This review will highlight important perspectives for future studies on the effectiveness of educational programmes focused on reducing the rate of sPIVC complications.

## Graphical abstract

**Figure:**
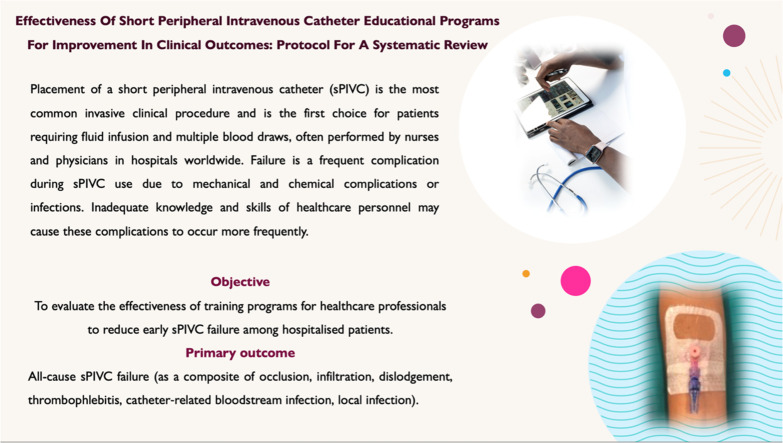

Specifications table

**Table:** 

Subject area:	Medicine and Dentistry
More specific subject area:	Nursing and Health Professions
Method name	Protocol for a systematic review
Name of your protocol:	Not applicable.
Reagents/tools:	Not applicable.
Experimental design:	Systematic review study
Trial registration:	Not applicable.
Ethics:	This study will not involve humans.
Value of the Protocol:	• The protocol may lead to developing evidence-based strategies that minimise catheter-related complications, resulting in better outcomes for hospitalised patients. • Finding new reliable estimates for the effectiveness of educational programmes focused on reducing vascular catheter-related complications in patients requiring a short peripheral intravenous catheter is crucial for informed clinical decision-making. • This protocol could lead to a better understanding of the impact of training programmes on sPIVC failure rates and complications, offering valuable information for future healthcare interventions and policies.

## Background

The placement of a short peripheral intravenous catheter (sPIVC) is the most common invasive clinical procedure, often performed by nurses and doctors in hospitals worldwide [1]. sPIVCs are the first choice solution for patients requiring fluid infusion and multiple blood draws [2]. However, sPIVC failure is frequently due to mechanical and chemical complications (i.e., phlebitis, dislodgement, occlusion, infiltration) or infections. Amongst sPIVC complications, phlebitis and infiltration are the most common. In a recent meta-analysis, the firsts occurred in 36% of 7323 patients, whereas the seconds occurred in 20.5% of 3638 patients when sPIVC were routinely replaced and in 23.9% of 3685 patients when they were replaced for clinical reasons [3]. Other complications, such as catheter occlusions and accidental removals, are less frequent, with a rate of 18.8% and 6.9%, respectively, while catheter-related bloodstream infections are rare (0.03–0.1%) [4,5].

A higher risk of complications was observed in patients with difficult intravenous access (DIVA), who have venous depletion due to conformational characteristics or particular medical conditions [7,8]. In these patients, the catheter is often placed at exit sites such as hands, wrists, ankles, feet, or external jugulars, which are at increased risk of patient discomfort and local complications, especially phlebitis and infiltration [9], [10], [11], [12].

Marsh et al. identified the occurrence of catheter-related complications as the main risk factors for the anticipated removal of sPIVC [6]. Furthermore, they observed that infiltrations increased significantly with the insertion of a 22 gauge sPIVC, compared to a 20 gauge, and that the risk of phlebitis was higher when the catheter was inserted in the dominant limb or when phlebitogenic drugs were administered [6]. Considering these risk factors as modifiable, the authors concluded that their early identification could reduce the high sPIVC failure rate.

Lack of knowledge of healthcare professionals was shown to be one of the main causes of sPIVC failure and short duration [13]. Previous studies showed that nurses' expertise in sPIVC placement, including the insertion site selection, catheter, way of fixation, dwell time, and the identification of treatment complications, should be improved [14,15]. Evidence suggests that implementing sPIVC placement skills increase the success rate of the first attempt, and showed fewer patients complications when procedures are performed by experienced nurses [13,16]. For this reason, implementing appropriate educational programmes to improve nursing expertise is crucial to providing comprehensive, evidence-based nursing care to patients [17,18]. Previous studies showed that developing a multimodal strategy and monitoring health care professionals with a regular evaluation of their adherence to hospital guidelines led to a sustained reduction in catheter-related infections [19,20].

The implementation of evidence-based practice in healthcare remains a complex challenge. The decision-making process should integrate evidence, settings, and stakeholders in a continuous and dynamic interaction [21]. Any training programmes should include strategies to promote the evaluation and reliability of clinical hospital guidelines, the experience of healthcare professionals, and the preferences of patients [22,23]. To our knowledge, no previous systematic reviews have been published to date evaluating the effectiveness of sPIVC management training programmes.

### Objective

This systematic review aims to evaluate the effectiveness of training programmes to reduce early sPIVC failure amongst hospitalised patients.

## Methods

This systematic review will follow the guidelines set out in the Cochrane Handbook for Systematic Review and Meta-Analyses [24]. The protocol has been registered in the PROSPERO database (ID: CRD42023444364). Review reporting will adhere to the Preferred Reporting Items for Systematic Reviews and Meta-Analyses (PRISMA) guidelines [25]. Deviations from the protocol will be recorded in the final report.

### Eligibility criteria

The research question was formulated using the PICO research question framework (*Participants*: healthcare professionals; *Intervention*: training programme; *Comparison*: No training programme; *Outcomes*: Infiltration, thrombophlebitis, occlusion, dislodgment, infections) [26].

#### Types of study

Randomised controlled trials will be included in the review. Prospective and retrospective observational studies, case series, and studies in which only the abstract is available will be excluded.

#### Types of participants

Studies including healthcare professionals responsible for sPIVC placement (physicians, nurses, midwives, etc.) working in wards caring for adult and paediatric patients will be included. Studies involving students and professionals still to be qualified will be excluded. No limits will be based on the work setting (e.g., emergency, long-stay, outpatient, etc.).

#### Types of intervention

Any training programme focused on sPIVC management will be included. No limits will be placed on educational programmes' duration or delivery method.

### Type of outcomes

#### Primary outcome

The primary outcome will be all-cause sPIVC failure (as a composite of infiltration, thrombophlebitis, occlusion, dislodgement, catheter‐related bloodstream infection, and local infection).

Vascular catheter-related complications were defined as follows:

• Infiltration is defined as the permeation of intravascular fluid into the interstitial compartment, causing tissue swelling around the catheter site [9];
• Thrombophlebitis (using any definition identified by the authors);
• Occlusion is the inability and/or impossibility to infuse fluids through the catheter due to an obstruction [27];
• Dislodgment: defined as catheter dislodgement (partial or complete) from the insertion site [6];
• Catheter‐related bloodstream infection (CRBSI), defined as a positive blood culture from a peripheral vein; clinical signs of infection; no other apparent source for the bloodstream infection except the IV catheter; and colonised IV catheter tip culture with the same organism as identified in the blood [3];
• Local infection (using any definition identified by the trial author).

#### Secondary outcome

• Pain during infusion (measured by any validated pain assessment scale);
• Satisfaction (measured by any validated satisfaction scale);
• Knowledge level (measured by a dedicated test, scale, score, etc.).
• Dwell time: defined as the time interval between the insertion and removal of the sPIVC [28].

### Search methods for the identification of studies

No limitations will be applied to the literature search. There will be no language-based exclusions of studies, and we will endeavour to translate studies published in languages other than English and Italian.

### Information sources

We will search PubMed, Embase, CINAHL, Cochrane Central Register of Controlled Trials (CENTRAL), the Cochrane Vascular Specialized Register through the Cochrane Register of Studies, http://clinicaltrials.gov/, http://www.who.int/ictrp/en/, http://www.controlled-trials.com/, and Google Scholar to identify relevant studies for review. For unpublished materials, such as conference proceedings, we will search databases of conferences proceedings and databases of the grey literature, such as MedNar and ProQuest. Bibliographic references from eligible studies will be evaluated to include any other work not found with the search strategy. We will also contact experts in the field to request any study we may miss from our database searches.

### Search strategy

We have developed a preliminary search strategy to identify relevant studies for review. The search strategy (*Supplementary materials*) was formed in PubMed and will be adapted to search other databases.

### Study records

#### Study selection process

Search results, including citation details and abstracts, will be entered into the dedicated software, and any duplicates will be automatically removed. Two authors will screen the titles and abstracts independently, and a third researcher will resolve any disagreements. The full texts of potentially relevant articles will be retrieved, and two authors will independently assess whether they are relevant to this review. A third evaluator will resolve any disagreement. The agreement between the two reviewers will be evaluated with Cohen's kappa coefficient. The agreement will be considered very good if equal to or greater than 0.8; good if between 0.61 and 0.80; moderate if between 0.41 and 0.60; fair if between 0.21 and 0.40; poor if K is less than 0.20 [29]. We will document the screening process using the PRISMA flow diagram [25].

#### Data extraction

We will extract the following main sets of data from each included study.

- First author, year of publication and country where the study was conducted (if available).
- Title of study;
- Aim of the study;
- Study population;
- Characteristics of the participants (gender and age);
- Study design, randomisation processes, allocation concealment;
- Description of the intervention evaluated (including details such as duration, intensity, content of the programme, method/mode of delivery, and who is delivering).
- Description of the intervention setting (hospital, home, residential aged care facilities);
- Numbers of participants in each trial arm, withdrawals, and dropouts;
- Outcome measures, time(s) at which outcomes were assessed;
- Funding source;
- Ethics approval and consent;
- Prospective registration in a clinical trials registry.

### Quality assessment of included studies

Two reviewers will assess the risk of bias in the included studies using the Cochrane Effective Practice and Organization of Care Risk of Bias tool for randomised controlled trials [30]. A third researcher will resolve any disagreements.

### Data synthesis

If the clinical and methodological components of the included studies are sufficiently similar and comparable, a meta-analysis will be performed when two or more studies investigate the same outcomes and interventions. We will analyse dichotomous outcomes by calculating the risk ratio (RR) for each included trial, along with the corresponding precision of the effect estimate expressed by their 95% confidence intervals. For continuous outcome measures, we expect to pool studies that use different scales and thus compute the standardized mean difference (SMD) and its corresponding 95% confidence interval for each included trial. Frequentist pairwise meta-analysis will be conducted considering fixed and random-effects models; heterogeneity will be investigated using the I^2^ and the corresponding p-value. Forest plots will be drawn to synthesise the results obtained for the comparisons of interest. If possible, funnel plots will be used to assess the potential for bias related to the size of the trials, which could indicate publication bias. The analysis will be performed using the software R 4.2.

### Assessing certainty

The certainty of the findings will be evaluated using the GRADE approach [31]. It provides four grades of evidence: very low, low, moderate, and high. Each result is given an initial quality ranking based on the study design and considering various factors such as the risk of bias, inconsistency, indirectness, imprecision, and publication bias from the studies examined. Depending on these factors, the ranking can either be lowered or elevated. To assess the overall body of evidence, we will develop a 'Summary of findings' tables for the following outcomes, using the GRADE approach: infiltration, thrombophlebitis, occlusion, and dislodgement.

## Discussion

Implementing evidence-based practice in healthcare remains a complex issue. This systematic review, the first on this topic, aims to establish the effectiveness of educational programmes in reducing early failure of sPIVC, such as infiltration, thrombophlebitis, occlusion, and dislocation, amongst hospitalised patients.  It will also highlight essential perspectives for future work on the effectiveness of educational programmes focused on reducing complications related to vascular catheter-related complications in patients requiring a short peripheral intravenous catheter.

## CRediT authorship contribution statement

**Daniele Privitera:** Conceptualization, Data curation, Investigation, Methodology, Writing – original draft, Writing – review & editing. **Erika Bassi:** Data curation, Investigation, Methodology, Writing – review & editing. **Chiara Airoldi:** Data curation, Investigation, Methodology, Writing – review & editing. **Nicolò Capsoni:** Data curation, Investigation, Methodology, Writing – review & editing. **Gloria Innocenti:** Data curation, Investigation, Methodology, Supervision, Writing – review & editing. **Isabella Santomauro:** Data curation, Investigation, Methodology, Supervision, Writing – review & editing. **Alberto Dal Molin:** Data curation, Investigation, Methodology, Supervision, Writing – review & editing.

## Declaration of Competing Interest

The authors declare that they have no known competing financial interests or personal relationships that could have appeared to influence the work reported in this paper.

## Data Availability

No data was used for the research described in the article.

## Appendix Supplementary materials

Image, application 1
