# Supplementary material for: Effectiveness of short peripheral intravenous catheter educational programmes to improve clinical outcomes protocol for a systematic review
Source: MethodsX. 2023 Aug 28;11:102352. doi: 10.1016/j.mex.2023.102352 (PMC10495618; doi:10.1016/j.mex.2023.102352)
Supplement: Supplementary file 1 [file mmc1.docx]

**Effectiveness of Short Peripheral Intravenous Catheter Educational Programs For Improving Clinical Outcomes: Protocol For A Systematic Review**

Daniele Privitera, RN, MSN^1,2^, Erika Bassi, RN, PhD^3^, Chiara Airoldi, MSc^3^, Nicolò Capsoni, MD^1^, Gloria Innocenti, BA^4^, Isabella Santomauro, RN, MSN^3^, Alberto Dal Molin, RN, PhD^3,5^

^1^ Department of Emergency Medicine, ASST Grande Ospedale Metropolitano Niguarda, Milan, Italy

^2^ Ph.D. Student, Department of Biomedicine and Prevention, University of Rome Tor Vergata, Italy.

^3^ Department of Translational Medicine, Università del Piemonte Orientale, Novara, Italy.

^4^ Centro Documentazione Biomedica, ASST Grande Ospedale Metropolitano Niguarda, Milan, Italy

^5^ Health Professions’ Direction, Maggiore della Carità Hospital, Novara, Italy.

**Address for correspondence:** Daniele Privitera, MSN, CCRN, Department of Emergency Medicine, ASST Grande Ospedale Metropolitano Niguarda, Milan, Italy. Tel +390264442294; e-mail: daniele.privitera@ospedaleniguarda.it

**SUPPLEMENTARY MATERIALS**

***MEDLINE STRING***

"health profession*"[Title/Abstract] OR nurs*[Title/Abstract] OR physician*[Title/Abstract] OR anaesthetist*[Title/Abstract] OR nurses[MeSH Terms] OR physicians[MeSH Terms] OR anesthetists[MeSH Terms] OR “Health Occupations”[MeSH Terms] OR “Health Occupation*”[Title/Abstract] AND "educational program*"[Title/Abstract] OR "educational course*"[Title/Abstract] OR "simulation program*"[Title/Abstract] OR "clinical education"[Title/Abstract] OR training*[Title/Abstract] OR simulat*[Title/Abstract] OR "learning program*"[Title/Abstract] OR "training program*"[Title/Abstract] OR course*[Title/Abstract] OR education*[Title/Abstract] OR education[MeSH Terms] OR trained[Title/Abstract] OR “computer simulation"[MeSH Terms] OR "computer simulation"[Title/Abstract] OR "Program Development"[MeSH Terms] AND Obstruct*[Title/Abstract] OR occlusi*[Title/Abstract] OR phlebitis*[Title/Abstract] OR infiltrate*[Title/Abstract] OR thrombophlebiti*[Title/Abstract] OR extravasat*[Title/Abstract] OR dislodgement[Title/Abstract] OR complication*[Title/Abstract] OR failure*[Title/Abstract] OR infection*[Title/Abstract] OR occlused[Title/Abstract] OR phlebitis[MeSH Terms] OR thrombophlebitis[MeSH Terms] OR "Extravasation of Diagnostic and Therapeutic Materials"[MeSH Terms] OR dislodge*[Title/Abstract] OR complication*[Title/Abstract] OR failure*[Title/Abstract] OR "Catheter-Related Infections"[MeSH Terms] OR "Catheter-Related Infections"[Title/Abstract] OR “CRBSI”[Title/Abstract] OR “BSI”[Title/Abstract] OR “bloodstream infection*”[Title/Abstract] OR “peripheral intravenous catheter failure”[Title/Abstract] AND (randomized controlled trial[Publication Type] OR (randomized[Title/Abstract] AND controlled[Title/Abstract] AND trial[Title/Abstract]))
